# Hepatocyte growth factor enhances death receptor-induced apoptosis by up-regulating DR5

**DOI:** 10.1186/1471-2407-8-325

**Published:** 2008-11-07

**Authors:** Yang Li, Xing Fan, C Rory Goodwin, John Laterra, Shuli Xia

**Affiliations:** 1 Hugo W. Moser Research Institute at Kennedy Krieger, Baltimore, MD, USA; 2 Department of Neurosurgery, University of Michigan Medical School, Ann Arbor, MI, USA; 3 Department of Oncology, Johns Hopkins School of Medicine, Baltimore, MD, USA; 4 Department of Neurology, Johns Hopkins School of Medicine, Baltimore, MD, USA; 5 Department of Neuroscience, Johns Hopkins School of Medicine, Baltimore, MD, USA

## Abstract

**Background:**

Hepatocyte growth factor (HGF) and its receptor c-MET are commonly expressed in malignant gliomas and embryonic neuroectodermal tumors including medulloblastoma and appear to play an important role in the growth and dissemination of these malignancies. Dependent on cell context and the involvement of specific downstream effectors, both pro- and anti-apoptotic effects of HGF have been reported.

**Methods:**

Human medulloblastoma cells were treated with HGF for 24–72 hours followed by death receptor ligand TRAIL (Tumor necrosis factor-related apoptosis-inducing ligand) for 24 hours. Cell death was measured by MTT and Annexin-V/PI flow cytometric analysis. Changes in expression levels of targets of interest were measured by Northern blot analysis, quantitative reverse transcription-PCR, Western blot analysis as well as immunoprecipitation.

**Results:**

In this study, we show that HGF promotes medulloblastoma cell death induced by TRAIL. TRAIL alone triggered apoptosis in DAOY cells and death was enhanced by pre-treating the cells with HGF for 24–72 h prior to the addition of TRAIL. HGF (100 ng/ml) enhanced TRAIL (10 ng/ml) induced cell death by 36% (*P *< 0.001). No cell death was associated with HGF alone. Treating cells with PHA-665752, a specific c-Met receptor tyrosine kinase inhibitor, significantly abrogated the enhancement of TRAIL-induced cell death by HGF, indicating that its death promoting effect requires activation of its canonical receptor tyrosine kinase. Cell death induced by TRAIL+HGF was predominately apoptotic involving both extrinsic and intrinsic pathways as evidenced by the increased activation of caspase-3, 8, 9. Promotion of apoptosis by HGF occurred via the increased expression of the death receptor DR5 and enhanced formation of death-inducing signal complexes (DISC).

**Conclusion:**

Taken together, these and previous findings indicate that HGF:c-Met pathway either promotes or inhibits medulloblastoma cell death via pathway and context specific mechanisms.

## Background

Hepatocyte growth factor (HGF) is a multifunctional cytokine that was originally described as a mesenchymal-derived factor that regulates cell growth, cell motility, morphogenesis and angiogenesis [1-3] through activation of its receptor, the transmembrane tyrosine kinase encoded by the *c-Met *proto-oncogene [4]. HGF and c-Met are often co-expressed or over-expressed in a variety of human malignancies including glioblastoma and medullablastoma; and their expression level correlates with poor prognosis [5-8]. The multifunctional effects of HGF:c-Met signaling in tumor cells are mediated by a network of signal transduction pathways including mitogen-activated protein kinase (MAPK) and phosphoinositide 3-kinase (PI3K). Paradoxically and dependent on cell context and the involvement of specific downstream effectors, both pro- and anti-apoptotic effects of HGF have been reported [9]. It is well documented that c-Met kinase-dependent signaling is able to counteract apoptosis induced by DNA-damage through the initiation of survival signals such as the PI3K-Akt, MAPK and NFκB pathways [10-13]. In addition, c-Met can bind to and sequester Fas via a kinase-independent mechanism in several types of cells, including epithelial and glioblastoma cells, and thereby prevent cell death induced by death receptor ligand [14,15]. On the other hand, the mechanisms by which HGF:c-Met exerts pro-apototic effects are not fully understood. It has been reported that HGF:c-Met signaling induces or sensitizes apoptotic cell death in a number of cell lines including ovarian carcinoma cell, breast carcinoma cell, mouse sarcoma cell and mouse hepatocarcinoma cell [16-19]. Although the anti-apoptotic functions of the HGF:c-Met pathway appear to predominate in most biological systems, pro-apoptotic responses have been observed and could contribute to the balance between cell death and survival during the initiation and progression of certain malignancies.

Embryonic neuroectodermal malignancies such as medulloblastoma are among the most common and aggressive childhood brain tumors, and are associated with high rates of morbidity and mortality. Significant improvements in survival have been achieved by treating patients early with combinations of radiation and chemotherapy (for reviews, see [20-22]). However, aggressive therapy during critical periods of CNS development results in considerable neurocognitive toxicity and durable responses in patients with recurrent medulloblastoma remain disappointing. Improving our understanding of medulloblastoma cell death and survival mechanisms and developing new strategies to overcome the inherent resistance of medulloblastoma cells to death signals could have significant impacts on survival and neurocognitive outcomes [23,24].

Induction of selective cancer cell death is the goal of many cancer therapies [25]. Apoptotic cell death can be initiated by either the intrinsic mitochondrial pathway or the extrinsic death receptor pathway [26]. Tumor necrosis factor (TNF)-related apoptosis-inducing ligand (Apo2L/TRAIL) has the potential to initiate death receptor pathways in malignant tissues while sparing normal tissues [27]. DR4 and DR5 are members of the TRAIL receptor superfamily sharing conserved cytoplasmic death domains essential for inducing apoptosis. Upon binding to TRAIL, DR4 and DR5 recruit the adapter molecule Fas-associated death domain (FADD), which through its death domain interacts with pro-caspases-8 and -10 to form a death-inducing signaling complex (DISC). DISC then stimulates the proteolytic activation of the caspases-8 and -10, initiating a cascade of downstream caspase activation which leads to apoptosis [28-30]. Nevertheless, TRAIL does not yet offer a viable solution to brain cancer therapy since many brain tumors display complete or partial-resistance to death receptor agonists [24,31,32].

Earlier work by our laboratory and others has shown that human medulloblastomas frequently express HGF, c-Met and molecular constituents of the extrinsic (death receptor) apoptosis pathway [7,33]. This study examines the interactions between HGF:c-Met signaling and medulloblastoma cell death/apoptosis in response to TRAIL. We found that in a representative medulloblastoma cell line, HGF promotes TRAIL-induced medulloblastoma cell death. We further examined the mechanism by which HGF exert pro-apoptotic effect. We demonstrate that the enhanced cell death response to TRAIL results from HGF-mediated DR5 over-expression and increased death-inducing signaling complex formation.

## Methods

### Reagents

All reagents were purchased from Sigma (St. Louis, MO) unless otherwise stated. Drugs were made in stock and diluted in cell culture medium. Recombinant human TRAIL was purchased from Millipore/Chemicon (Billerica, MA). Hepatocyte growth factor (HGF) was a gift from Genentech, Inc. (San Francisco, CA). The JNK pathway inhibitor CEP-11004 was kindly provided by Cephalon Inc. (West Chester, PA) [34]. The c-Met kinase inhibitor PHA-665752 was kindly provided by Pfizer, Inc. (La Jolla, CA) [35]. Caspase 3, 8, 9 inhibitors were purchased from Calbiochem (San Diego, CA).

### Cell culture

Medulloblastoma cell line DAOY was originally purchased from American Type Culture Collection (Rockville, MD). DAOY cells were grown in Zinc Optimum medium (Gibco, Rockville, MD) supplemented with 10% fetal bovine serum (FBS, Cellegro, Washington, DC) and 500 μg/ml penicillin-streptomycin (Gibco). Cells were grown at 37°C in a humidified incubator with 5% CO_2_.

### Cell viability assay

Cell viability was measured by MTT (3-(4,5-dimethylthiazol-2-yl)-2,5-diphenyl tetrazolium bromide) assay. Cells were plated at 5,000 cells/well in 24-well tissue culture plates. After treatment, MTT was added to each well at a final concentration of 150 μg/mL, and the cells were incubated for 1–2 h at 37°C. The medium was then removed, and the cell layer was dissolved with dimethyl sulfoxide (DMSO). The formazen reaction product was quantified spectrophotometrically at 570 nm using a Spectra MAX 340pc plate reader (Molecular Devices, Sunnyvale, CA, USA). The results are expressed as the percentage of absorbance measured in control cultures after subtracting the background absorbance from all values.

### Flow cytometry analysis

Apoptosis was quantified using the Annexin V-FITC apoptosis detection kit (BD Biosciences, San Diego, CA) via the manufacturer's instructions. Briefly, cells were trypsinized, pelleted by centrifugation, and resuspended in Annexin V binding buffer (150 mM NaCl, 18 mM CaCl_2_, 10 mM HEPES, 5 mM KCl, 1 mM MgCl_2_). FITC-conjugated Annexin V (1 μg/ml) and propidium iodide (PI, 50 μg/ml) were added to cells and incubated for 30 min at room temperature in the dark. To quantify cell surface DR4 and DR5, cells were harvested by trypsinization and centrifugation. The suspended cells were directly incubated with PE-conjugated anti-DR4 or anti-DR5 antibody for 30 min (1:20, EBiosciences, San Diego, CA). Non-immune mouse IgG was used as the negative control. Analyses were performed on a FACscan (Becton-Dickinson, Mountain View, CA). Data were analyzed with CellQuest software (Becton-Dickinson).

### Western blot analysis

All primary antibodies used for Western blot analysis were obtained from Cell Signaling Technology (Beverly, MA) unless otherwise stated and concentrations used were according to the manufacturers' recommendations. All the secondary antibodies conjugated to horseradish peroxidase were purchased from Jackson Immunoresearch Laboratories (West Grove, PA) and were used at a 1:1000 dilution. Briefly, cells were lysed with RIPA buffer (50 mM Tris-HCl, pH 7.4, 150 mM NaCl, 1% NP-40, 0.25% Na-deoxycholate) containing 1× protease and phosphatase inhibitor cocktail (Calbiochem). After sonication for 15 s, the suspensions were centrifuged at 3,000 *g *for 10 minutes. Protein concentrations were determined using the Coomassie Protein Assay Reagent (Pierce, Rockford, IL). Thirty micrograms of protein were subjected to 4%–20% gradient SDS-PAGE (Lonza, Pittsburgh, PA) and then transferred to nitrocellulose membranes (Amersham, Pittsburgh, PA) for 1 h. The membranes were then incubated with 5% non-fat dry milk (Carnation, Nestle Food Co., Glendale, CA) in Tris-Buffered Saline Tween-20 (TBST) for 1 h, and then incubated with primary antibody overnight at 4°C in either 5% non-fat dry milk or in 5% BSA. Membranes were subsequently rinsed in TBST, and then incubated for 1 hour at room temperature with secondary antibody conjugated with horseradish peroxidase. After incubation, membranes were rinsed, and antibody binding was detected with the enhanced chemiluminescence system (Amersham).

### Immunoprecipitation

Cell pellets were treated with lysis buffer (100 mM Tris-HCL, 5 μM EDTA and 1% NP-40) containing 1× protease inhibitor Cocktail and 1× Phosphatase inhibitor cocktail. Protein concentrations were determined as above. Total protein (500 μg) were incubated with 5 μg anti-DR5 antibody (Axxora, San Diego, CA) at 4°C for 1 h. Protein A/G beads (Santa Cruz, San Diego, CA) were added to the lysate and incubated overnight at 4°C with gentle rocking. The beads were washed 5 × by repeated suspension in 500 μl lysis buffer followed by centrifugation at 10,000 g for 30 s. After the last wash, 50 μl Laemmli buffer (4% SDS, 20% glycerol, 10% 2-mercaptoethanol, 0.004% bromphenol blue, 0.125 M Tris HCl, pH 6.8) was added to the pellet and heated at 100°C for 10 min. The samples were centrifuged again to collect supernatant and then subjected to Western blot analysis as described above.

### Northern blot analysis

Northern hybridization was used to examine the changes in DR5/DR4 expression level in DAOY cells after HGF treatment. RNA was isolated using the RNeasy Mini Kit (Qiagen, Valencia, CA) following the manufacturer's directions. Oligonucleotide primers were designed as follows: for DR5 (NM_003842), a 587 bp product was amplified with primer pair 5'-caccacgaccagaaacacag/5'-gcctcctcctctgagacctt; for DR4, a 569 bp product was amplified with primer pair 5'-gctgcaaccatcaaacttca/5'-ttgtgagcattgtcctcagc. RT-PCR was conducted using DAOY RNA as a template and PCR products were subcloned in TOPO PCR cloning vectors (Invitrogen, Carlsbad, CA) and sequenced prior to use. Northern blot analysis was performed with ^32^P-labeled cDNA probes as previously reported [36]. Radioactivity was quantified by densitometry and by phosphorimaging using the Bio-Imaging analyser BAS 2500 (Fuji Medical Systems; Stamford, CT). All blots were stripped in 1 × saline sodium citrate buffer (SSC)/0.1% sodium dodecyl sulfate (SDS) maintained at a temperature of 85°C for 10 min and then rehybridized with cDNA specific for 28S rRNA. Results are expressed relative to 28S rRNA.

### Quantitative reverse transcription-PCR (RT-PCR)

Total RNA was extracted from snap-frozen tumor tissue using Trizol reagent (Invitrogen) via manufacturer's instructions. RNA was then treated with DNase and further purified using the RNeasy protocol (Qiagen). RT-PCR for tumor necrosis factor receptor superfamily, member 10b (DR5) was done as previously described [37], with all reactions normalized to actin (Applied Biosystems, Foster City, CA). Commercially available Assay on Demand TaqMan primers and probes were used to measure mRNA for DR5 (catalog number 4331182). Serial dilution of total RNA from DAOY was used to generate standard curves. The expression of c-Met and DR5 in all clinical samples was calculated in relation to these. Each quantitative RT-PCR reaction was done in triplicate and error bars represent SE.

### Statistical analysis

Statistical analyses consist of one-way ANOVA followed by the Tukey multiple comparison tests using Graphpad Prism (GraphPad, San Diego, CA). Log Pearson analysis (Graphpad Prism) was used to determine the correlation between c-Met and DR5 expression in human embryonal CNS tumors. All experiments reported represent at least three independent replications. Data are represented as mean value ± standard error of mean (SE).

## Results

### HGF enhances TRAIL-induced apoptosis in medulloblastoma cells

**Figure 1:**
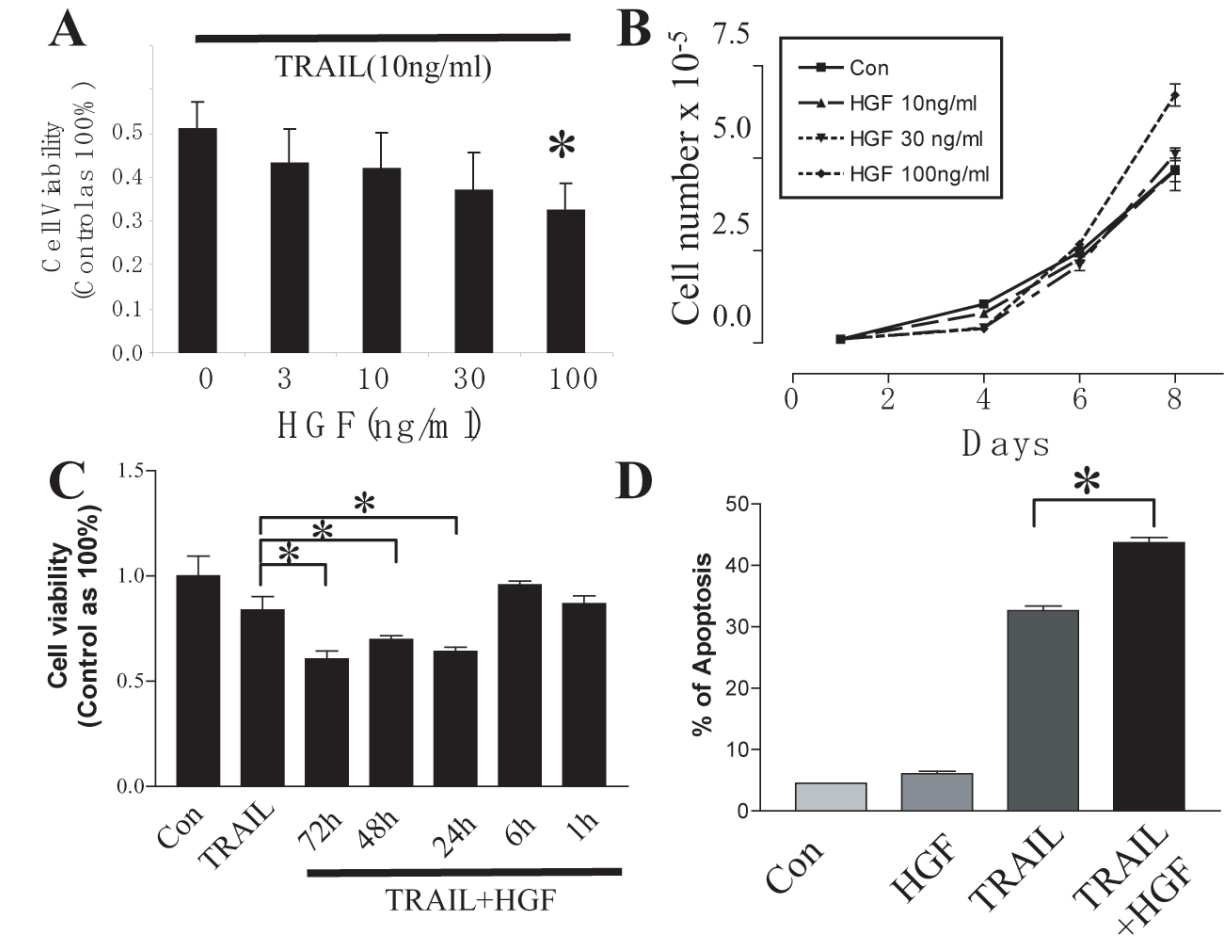
**HGF potentiates TRAIL induced apoptosis in DAOY cells**. **A**. HGF enhances TRAIL induced cell death in a dose-dependent manner as measured by MTT assay. TRAIL at 10 ng/ml induced 50% cell loss. Pre-incubation with HGF (100 ng/ml) for 72 h resulted in 18% more cell death compare to TRAIL alone. **B**. HGF alone induces DAOY cell growth. Cells were counted every two days after plating with or without HGF in medium containing 10% FBS. HGF promoted cell growth in a dose-dependent manner. **C**. HGF sensitizes TRAIL (3 ng/ml) induced cell death in a time-dependent manner. Pre-incubation with HGF for at least 24 h was required to enhance TRAIL-induced cell death. **D**. FACscan analysis of DAOY cell apoptosis using Annexin V-FITC and propidium iodide (PI) staining. Annexin V-positive cells were found to be 6%, 32% and 44% in the presence of HGF (100 ng/ml), TRAIL (10 ng/ml) and HGF+TRAIL, respectively. Experiments were repeated three times. Data represents mean ± SE (N = 9, * *P *< 0.001).

In the DAOY human medulloblastoma cell line, we found that HGF sensitizes TRAIL induced apoptotic cell death. Incubating cells with TRAIL alone (10 ng/ml) for 24 h induced death in 50% of cells (Fig. 1). HGF (100 ng/ml) together with TRAIL (10 ng/ml) induced 68% cell death. Thus, HGF increased the cell death response to TRAIL by 36% (Fig. 1A, *P *< 0.001, TRAIL+HGF *vs *TRAIL alone). No cell death was observed in response to HGF alone. In fact, incubating DAOY cells with HGF alone (100 ng/ml) for 8 days increased cell numbers by ~40% (Fig. 1B). This is consistent with our previous observations that HGF induces cell cycle progression in medulloblastoma cells including the DAOY cell line [7]. The enhancement of cell death required that cells be treated with HGF for at least 24 h prior to the addition of TRAIL. Shorter HGF pre-treatment time ranging from 1–6 h failed to significantly enhance TRAIL induced cell death (Fig. 1C). Thus, HGF promotes TRAIL induced cell death in a concentration- and time-dependent manner.

Annexin V-FITC cell staining with flow cytometry analysis was used to determine if HGF sensitizes DAOY cells to apoptosis. HGF alone (100 ng/ml) did not alter cell viability (6% annexin V positive cells). Treating cells with 10 ng/ml TRAIL for 24 h increased the proportion of annexin V positive cells to 32%. Treating cells with HGF for three days prior to TRAIL treatment increased annexin V positive cells to 44% (Fig. 1D. *P *< 0.001 TRAIL+HGF *vs. *TRAIL alone), confirming that HGF sensitize these cells to TRAIL-induced apoptosis.

### Apoptotic pathways activated by TRAIL + HGF

**Figure 2:**
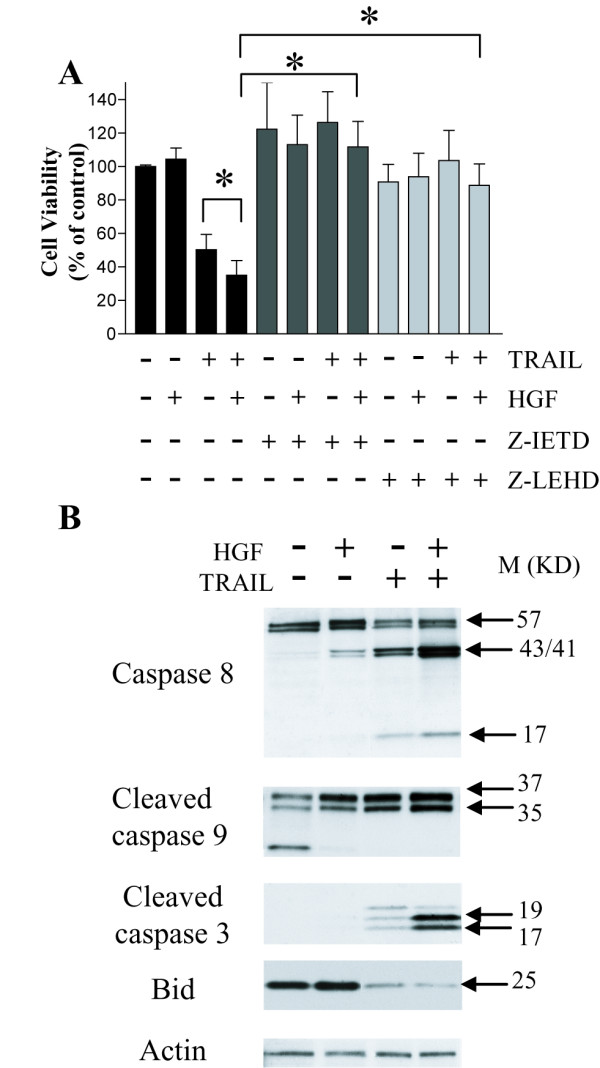
**Involvement of intrinsic and extrinsic apoptotic pathways in cell death induced by TRAIL + HGF**. **A**: MTT assay showing cytoprotection from specific inhibitors of caspase-8 (Z-IETD, 50 μM) and caspase-9 (Z-LEHD, 50 μM) against TRAIL + HGF induced apoptosis. DAOY cells were pre-incubated with HGF for 72 h and then treated with caspase inhibitors for 30 min before adding TRAIL. Z-IETD reversed cell death induced by TRAIL + HGF completely, whereas Z-LEHD protected ~80% of the cells from death. **B**. Western blot analysis of caspase-8, -9, -3 and Bid. Cells were treated with or without HGF (100 ng/ml) for 72 h before incubation with TRAIL (10 ng/ml) for another 24 h. TRAIL alone induced activation (cleavage) of caspase-3, 8, 9 and Bid. HGF enhanced the activation of caspases and Bid by TRAIL. Experiments were repeated three times (N = 9, * *P *< 0.001).

We examined the apoptotic pathways induced by TRAIL + HGF. Cell death induced by TRAIL+HGF was significantly reversed by either the caspase-8 inhibitor (Z-IETD-FMK, 50 μM) or the caspase-9 inhibitor (Z-LEHD-FMK, 50 μM) (Fig. 2A). This implicated the involvement of both extrinsic and intrinsic apoptosis pathways in the cell death response. This result was substantiated by the increased activation of caspase-3, caspase-8, and caspase-9 in cells treated with both TRAIL + HGF relative to cells treated with TRAIL alone (Fig. 2B). Furthermore, cleavage of the pro-apoptotic protein Bid, as evidenced by a decrease in full-length Bid, was greatest in cells treated with TRAIL + HGF (Fig. 2B).

### Signaling pathways involved in HGF enhanced cell death

**Figure 3:**
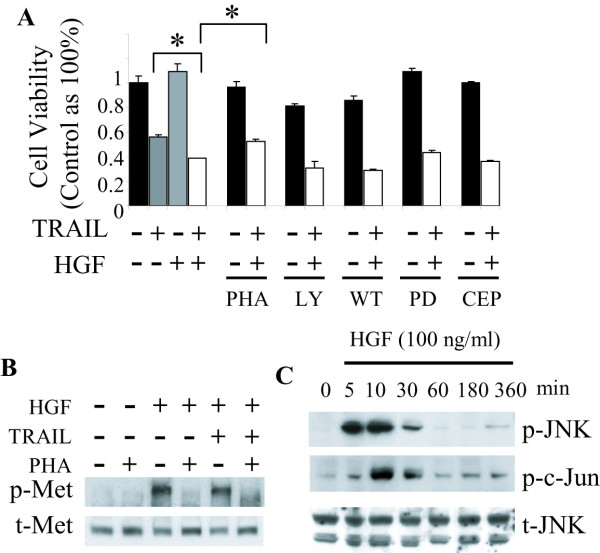
**Signaling pathways mediating the potentiation of TRAIL-induced cell death by HGF**. **A**. DAOY cells were pre-incubated with or without inhibitors for 30 min, and then treated with HGF for 72 h followed by TRAIL for 24 h. Cell viability was analyzed by MTT assay. The c-Met inhibitor PHA-665752 (100 nM) significantly reduced the cell death promoting effect of HGF. The PI3 kinase inhibitor LY294002 (30 μM) or wortmannin (1 μM) slightly increased the cell death rate induced by TRAIL + HGF. The ERK inhibitor PD98059 (30 μM) as well as the JNK pathway inhibitor CEP-11004 (10 μM) had no effect on the cell death induced by TRAIL + HGF. **B**. DAOY cells were pre-incubated with c-Met inhibitor PHA-665752 (100 nM) for 30 min before adding HGF. Phosphorylation of c-Met in cells treated with HGF or HGF+TRAIL was inhibited by PHA-665752. **C**. Western blot analysis with anti-JNK and anti-phospho-JNK (Ser 198) antibodies was used to determine JNK activation. In DAOY cells, HGF induced JNK activation as early as 5 min and lasted for 30 min. C-Jun was also activated within 10 min of HGF treatment. Data represent mean ± SE (N = 9, * *P *< 0.001).

C-Met has the potential to complex with and modulate death receptors independent of its classical tyrosine kinase driven second messenger responses [14,15]. Therefore, we asked if the cell death promoting effects of HGF requires c-Met tyrosine kinase activity. Inhibition of c-Met activation with PHA-665752 (100 nM) significantly abrogated the enhancement of TRAIL-induced cell death by HGF (Fig. 3A). Thus, the death promoting effect of HGF requires activation of the c-Met kinase. Western blot analysis confirmed that c-Met was activated as early as 5 minute after treating DAOY cells with HGF (Fig. 3B) and this is completely inhibited by PHA-665752.

AKT and ERK are activated downstream of c-Met and are required for maximizing HGF-induced cell proliferation and protection against DNA damage agents [7,33]. We asked if inhibiting these pathways alters the death enhancing effects of HGF in DAOY cells. The effects of specific PI3 kinase inhibitors LY294002 (30 μM) and wortmannin (1 μM) and the specific ERK inhibitor PD98059 (30 μM) on cell death induced by TRAIL + HGF were examined. LY294002 and wortmannin alone slightly induced cell death (80% and 85% cell survival, respectively, vs. control 100%). Both of them increased cell death induced by HGF+TRAIL (30% and 29% cell survival, respectively, vs 38% in control), though not statistically significantly. Treating cells with PD98059 did not affect cell viability under control conditions, and did not change the magnitude of cell death induced by TRAIL+HGF (43% cell survival vs 38% in control, Fig. 3A).

C-jun NH2-terminal kinase (JNK) is also activated by HGF and contributes to cell death promoted by HGF in several models [38]. We also examined if JNK is activated by HGF in DAOY cells and whether JNK activation is required to sensitize DAOY cells to TRAIL. JNK was strongly activated by HGF, reaching maximal activation as early as 5–10 minutes after HGF addition (Fig. 3C). C-Jun, the down-stream target of JNK, also showed a similar temporal pattern of activation following HGF treatment, reaching maximal activation in 10 minutes (Fig. 3C). However, the activated phosphorylated forms of JNK and c-Jun diminished rapidly, returning to baseline ~1 h after HGF exposure. Pre-incubating cells for 24 h with the specific JNK pathway inhibitor CEP-11004 (10 μM) under conditions that inhibit JNK phosphorylation by ~80% [39] did not attenuate cell death induced by HGF+TRAIL (Fig. 3A).

### DR5 up-regulation by HGF

**Figure 4:**
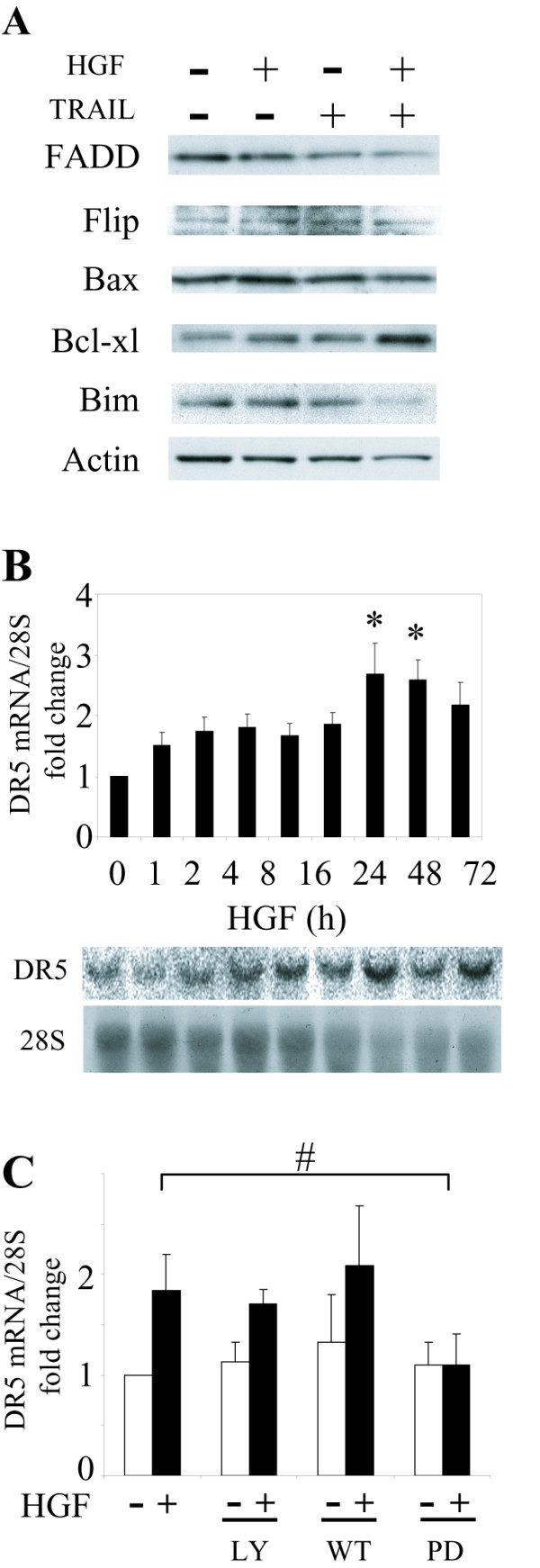
**Modulation of pro- and anti-apoptotic proteins by HGF**. **A**. Western blot analysis of FADD, FLIP, Bax, Bcl-xl and Bim protein levels in response to HGF, TRAIL and HGF + TRAIL. Cells were treated with HGF for 72 h before incubation with TRAIL for another 24 h. FADD and Bim were slightly down regulated by HGF + TRAIL. **B**. Northern blot analysis of DR5 mRNA levels in response to HGF. DAOY cells were treated with HGF for the indicated times. Total cell RNA was hybridized with probes for DR5 and 28S RNA (control) as described in Materials and Methods. DR5 isoform 1 mRNA levels were increased between 1–72 h as shown (* *P *< 0.001). **C**. DAOY cells were pre-incubated with inhibitors for 30 min, and then treated with HGF for 24 h followed by Northern blot analysis for DR5 expression. The PI3K inhibitor LY294002 (30 μM) or wortmannin (1 μM) did not affect the DR5 up-regulation induced by HGF. The MAPK inhibitor PD98059 (30 μM) reversed up-regulation of DR5 by HGF (# *P *< 0.05). Data represent mean ± SE (N = 6).

The effects of HGF on key regulatory components of the extrinsic and intrinsic apoptotic pathways were examined by Western blot analysis. To summarize, total cell levels of the pro-apoptotic protein FADD were diminished slightly by TRAIL in both control and HGF-treated cells (0.9 and 0.85-fold, respectively). Levels of pro-apoptotic Bim were decreased by TRAIL in HGF-treated cells only (0.55-fold). The anti-apoptotic protein Bcl-xl was found to be substantially up-regulated by TRAIL in HGF-treated cells (1.62-fold). There was no significant change in Bax or FLIP protein levels (Fig. 4A). Bcl-2 and Bad were not detected in DAOY cells (data not shown). Thus, HGF does not appear to sensitize cells to TRAIL by affecting levels of these specific anti- or pro-apoptotic proteins.

We examined the effect of HGF on the expression of TRAIL receptors DR4 and DR5. Two isoforms of the DR5, tumor necrosis factor receptor superfamily member 10b (TNFRSF10B) gene, have been sequenced in humans. Northern blot analysis showed a time-dependent increase in DR5 isoform 1 mRNA expression beginning as early as 1 h after the addition of HGF and reaching a maximum of ~2.5 fold over baseline at 24 h. DR5 isoform 1 mRNA levels remained ~2 fold above baseline 3 days after treating cells with HGF (Fig. 4B). Isoform 2, which contains a 19 amino acid deletion, was not detected by Northern hybridization (data not shown).

**Figure 5:**
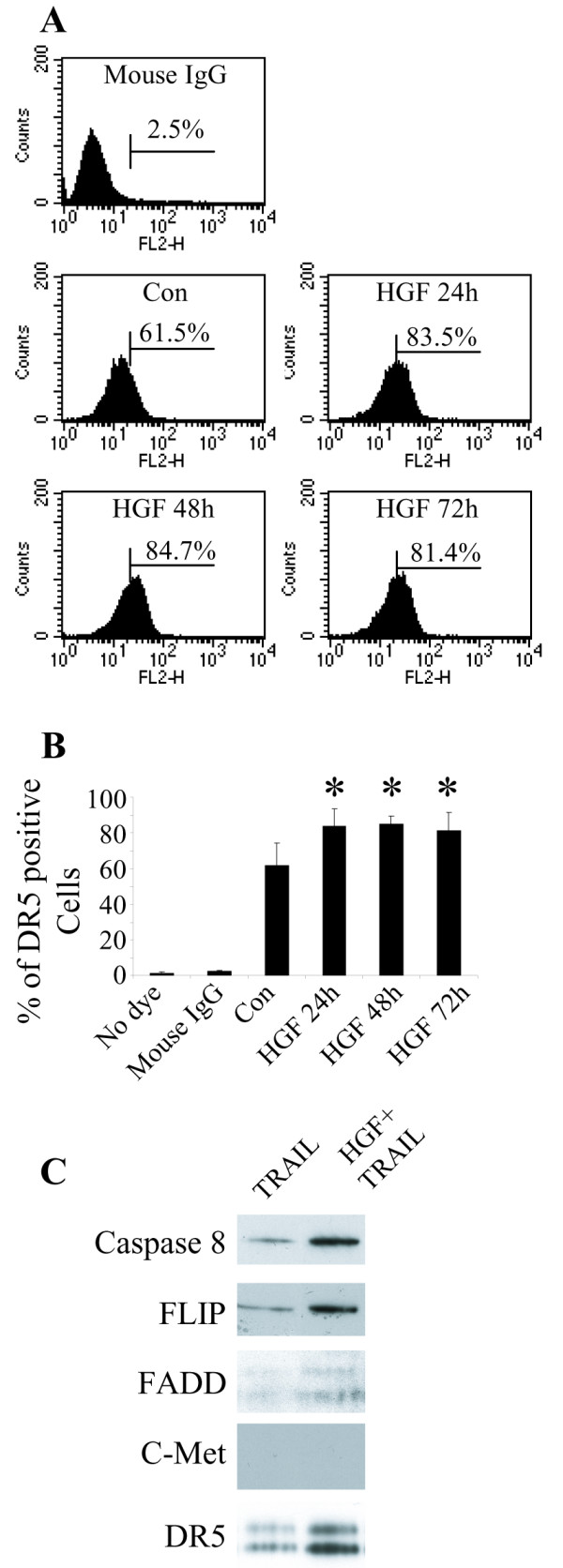
**HGF increases the DR5 expression and enhances TRAIL-induced DISC formation**. **A**. FACScan analysis of DR5 expression in DAOY cell surface after HGF treatment. Cells treated with HGF for 24 – 72 h were incubated with PE-conjugated anti-DR5 antibody for 30 min. Mouse IgG was used as negative control. At baseline ~61.5% cells were DR5 positive. After 24 h incubation with HGF, the DR5 positive pool increased to 83.5%. **B**. Compared to control, there was a significant increase in DR5 positive cells after 24 – 72 h incubation with HGF (N = 6, * *P *< 0.05). **C**. DAOY cells were pre-treated with HGF for 72 h followed by TRAIL for 2 h. Equal amounts of total cell protein (500 μg) were immunoprecipitated with anti-DR5 antibody and then subjected to Western blot analysis with antibodies to caspase 8, FLIP, FADD, c-Met and DR5 antibody. There was a distinct increased in DISC in cells treated with TRAIL + HGF.

DR5 up-regulation in response to HGF was further examined at the protein level by flow cytometry. Cells were stained with anti-DR5 antibody conjugated with PE and subjected to flow cytometric analysis. The DR5-positive cell fraction rose from 61.5% to 83.5% after treating cells with HGF for 24 hours and the DR5-positive fraction remained elevated 72 h after treatment (Fig. 5A, B). There was no or very little expression of DR4 protein in DAOY cells as detected by flow cytomery; and HGF did not change the expression of DR4 (data not shown).

We asked if inhibiting HGF:c-Met downstream signaling pathways including PI3K-AKT and MAPK alters the HGF-mediated DR5 increase in DAOY cells. Cells were pre-incubated with different inhibitors for half hour followed by treatment with HGF for 24 hours. DR5 expression was measured by Northern blot analysis as mentioned above. The PI3K-AKT inhibitors LY294002 (30 μM) and wortmannin (1 μM) did not affect the DR5 up-regulation induced by HGF. However, the ERK inhibitor PD98059 (30 μM) blunted up-regulation of DR5 (Fig. 4C, *P *< 0.05 vs. HGF). This suggests that MAPK pathway may play a role in HGF-mediated DR5 up-regulation.

We further asked if HGF alters death-inducing signal complex (DISC) formation. DAOY cell protein extracts obtained 2 h following TRAIL treatment were subjected to immunoprecipitation with anti-DR5 antibody; precipitated complexes were then analyzed by Western-blot using antibodies against caspase 8, Flip, FADD and DR5. All of these DISC components were increased in HGF + TRAIL treated cells (Fig. 5C). In contrast, c-Met was not co-precipitated with DR5, indicating no direct interaction between c-Met and DR5 (Fig. 5C), further supporting our finding that c-Met enhances DR5 expression at the transcriptional level.

### Expression of DR5 and c-Met in human embryonal tumors

**Figure 6:**
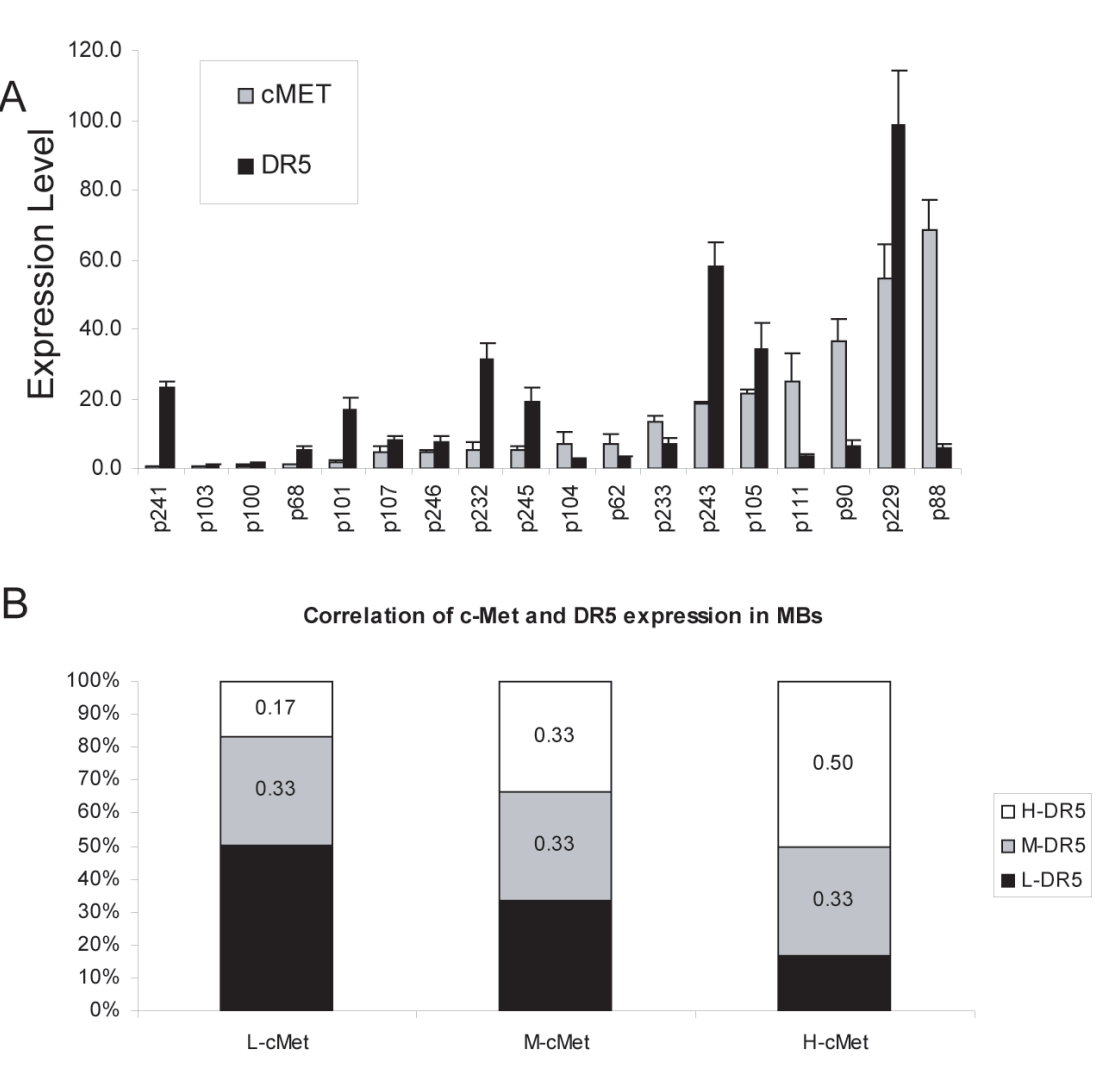
**Relationship between c-Met and DR5 expression in human embryonal CNS tumors**. **A**. Quantitative reverse transcription-PCR results showing the expression levels of c-Met and DR5 relative to actin in 18 snap-frozen tissues samples. The expression of c-Met and DR5 in all samples was calculated in relation to DAOY expression. **B**. We assigned tumors to either high (upper third), median (middle third) and low (lowest third) c-Met expression or TRAIL expression (N = 6 per group). Of the 6 tumors expressing high c-Met (H-cMet), 50% expressed high DR5. Of the 6 tumors expressing low c-Met (L-cMet), 50% expressed low level of DR5. The correlation between c-Met expression and DR5 expression is 0.4 (*P *= 0.06, Log Pearson analysis).

Moreover, we asked if DR5 increase by HGF in the DAOY cell line reflects a relationship between DR5 and the HGF:c-Met pathway in human embryonal CNS tumors. RT-PCR was used to measure the levels of c-Met and DR5 mRNA in 18 snap-frozen human embryonal tumor specimens (14 medulloblastoma and 4 peripheral neuroectodermal tumors (PNET)). Of the 18 tumor samples tested, all contained detectable levels of c-Met and DR5 mRNA. The expression level of c-Met is from 0.5 to 68.4 (c-Met/actin, normalized to DAOY). The expression level of DR5 in the 18 tumor samples ranges from 1.0–98.5 (DR5/actin, normalized to DAOY). In normal brain tissue the expression level of c-Met and DR5 is 0.45 and 2.4, respectively. DR5 and c-Met do show a moderate positive correlation (r = 0.4, *P *= 0.06, Log Pearson analysis) (Fig. 6A). This relationship is best depicted after the 18 tumor specimens were independently divided into three groups each containing 6 tumors based on c-Met expression levels (highest, middle, and lowest thirds) and similarly into three groups based on DR5 expression levels. Of the 6 tumors with highest c-Met expression, 50% were among the highest and 17% were among the lowest DR5 expressers, respectively. In contrast, of the 6 tumors with lowest c-Met expression, 17% were among the highest and 50% were among the lowest DR5 expressers, respectively (Fig. 6B). This trend suggests that HGF:c-Met pathway activity influences DR5 expression and may in turn influence TRAIL induced cell death in human embryonal cell malignancies.

## Discussion

We show that HGF promotes TRAIL-induced cell death in the human medulloblastoma DAOY cell line. We implicate a mechanism by which activating the c-Met tyrosine kinase enhances DR5 death receptor expression, resulting in enhanced DISC formation and downstream effector caspase-dependent apoptosis in response to TRAIL. This is, to the best of our knowledge, the first study demonstrating up-regulation of DR5 by the HGF:c-Met signaling pathway. We also identify a relationship between c-Met and DR5 expression level in clinical brain tumor specimens, suggesting that our in vitro findings may be translatable to a subset of human embryonal CNS tumors.

HGF is well known for its ability to protect tumor cell death triggered by various DNA-damaging stimuli including gamma-radiation and chemotherapeutic agents [10-13]. We reported previously that HGF protects DAOY medulloblastoma cells against chemotherapy-induced apoptosis [7]. MacDonald and colleagues have compared the gene expression patterns of medulloblastoma cell lines to medulloblastoma clinical specimens and concluded that DAOY cells most closely mimic metastatic medulloblastoma, supporting the potential relevance of our in vitro results [40]. The potential that our apoptosis-promoting results might be more broadly applicable to human medulloblastoma is further supported by the correlation between c-Met and DR5 expression level in human embryonal CNS tumors. The cell specific molecular determinants of pro- and anti-apoptosis responses to HGF:c-Met pathway activation are of interest and will require further investigation.

The second messenger pathways downstream of c-Met activation are multifunctional and can include cytoprotection, proliferation or death promotion [11,14,36,38]. The PI3K-AKT pathway, which is potently activated by HGF, mediates cell protection in a large number of in vitro models. In DAOY cells, the overall effect of c-Met activation was to enhance TRAIL-induced death. We used the PI3K specific inhibitors LY294002 and wortmannin to specifically examine the role of PI3K-AKT pathway activity in the sensitivity of HGF-treated DAOY cells to TRAIL. While not statistically significant, we found that both inhibitors increased TRAIL-induced cell death in HGF-treated cells. Thus, it is possible that PI3K-AKT pathway activation by HGF:c-Met signaling does have cytoprotective actions against in TRAIL-induced DAOY cell apoptosis even though the predominant effect of c-Met activation is death promoting under our experimental conditions. Things become more complicated when the cells were pre-treated with the MAPK specific inhibitor PD98059. Similar as PI3K-AKT, MAPK play an important role in cytoprotection (6, 7). Our Northern blot analysis data showed that MAPK may also mediate the DR5 up-regulation induced by HGF. The two aspects of MAPK may counteract and this may explain that the net effect of PD98059 is neutral. The JNK (c-jun NH2-terminal kinase), a well known cell death promoting signaling pathway, was found to be activated by HGF and to mediate HGF-induced cell death in other tumor models [38]. We found that in DAOY cells, JNK was transiently activated by HGF, peaking within 1 hour, and then quickly returned to base line. It has been postulated that sustained JNK activation is required to activate death-promoting downstream targets such as Bim and Bak [41]. The fact that JNK was only transiently activated by HGF is consistent with at most a minor role of JNK in sensitizing DAOY cells to TRAIL induced apoptosis. The fact that the JNK pathway specific inhibitor CEP-11004 failed to reverse the death promoting effect of HGF further supports the notion that sustained JNK activation is required to promote apoptosis.

C-Met activation by HGF can inhibit Fas-induced hepatocyte apoptosis by preventing the degradation of FLIP_*L *_in response to activated Fas [15]. In contrast to this, we found that c-Met activation does not modulate FLIP_*L *_levels in DAOY cells treated with TRAIL. Whether or not HGF affects FLIP function via other mechanisms will require further investigation. Complex formation between c-Met and the death receptor Fas has been found in other cell lines [14,19]. In these reports, the interaction between c-Met and Fas was found to inhibit DISC formation and thereby protected cells from apoptosis. Based on these reports, it is possible that HGF might dissociate c-Met from TRAIL receptors and thereby promote TRAIL-induced cell death [19]. However, co-immunoprecipitation experiments did not detect complexes with c-Met and DR5, the predominant TRAIL receptor expressed by DAOY cells. Instead, HGF was found to upregulate DR5 expression and increase the formation of death-inducing signaling complexes containing DR5 and caspase-8. Overall, our findings suggest that DR5 enhancement is the predominant mechanism by which HGF potentiates TRAIL-induced cell death in DAOY cells.

## Conclusion

In conclusion, we have demonstrated for the first time that c-Met kinase activity can up-regulate death receptor expression and thereby sensitize tumor cells to the death receptor ligand TRAIL. Our analysis of clinical specimens suggests a correlation between c-Met and DR5 expression level in a subset of human embryonal CNS malignancies. These findings extend the notion that the c-Met receptor tyrosine kinase can have dual anti-apoptotic and pro-apoptotic actions in a common pediatric brain malignancy. Our findings suggest that death receptor activation may provide a route to overcome chemo/radio-resistant cells in subsets of embryonal CNS malignancies that express an activated c-Met pathway.

## Competing interests

The authors declare that they have no competing interests.

## Authors' contributions

YL conducted most of the experiments in this study. XF contributed Fig. 6. CRG contributed part of Fig. 1. JL was responsible for the overall design of the study, and edited the manuscript. SX initiated the study and drafted the manuscript. All authors read and approved the manuscript.

## Pre-publication history

The pre-publication history for this paper can be accessed here:


